# A randomized controlled trial evaluating prophylactic treatment of *Artemisia* pollinosis using azelastine hydrochloride and fluticasone propionate nasal spray

**DOI:** 10.3389/falgy.2025.1559201

**Published:** 2025-04-10

**Authors:** Le Cui, Na Gao, Cairong Bai, Yali Zuo, Wendong Hao, Kai Guan

**Affiliations:** ^1^ Department of Allergy, Peking Union Medical College Hospital, Chinese Academy of Medical Sciences & Peking Union Medical College, Beijing, China; ^2^ Beijing Key Laboratory of Precision Medicine for Diagnosis and Treatment on Allergic Diseases, Peking Union Medical College Hospital, Beijing, China; ^3^ National Clinical Research Center for Dermatologic and Immunologic Diseases, Beijing, China; ^4^ Department of Allergy, The First Affiliated Hospital of Xi’an Jiaotong University, Yulin Hospital, Yulin, China

**Keywords:** randomized controlled trial, prophylactic treatment, *Artemisia*, pollinosis, allergic rhinitis, asthma

## Abstract

**Background:**

Prophylactic treatment for pollinosis is advantageous for managing nasal symptoms in patients with seasonal allergic rhinitis. Inadequate control of rhinitis symptoms increases the risk of acute asthma attacks. Nevertheless, there is limited research on the use of nasal glucocorticoids and antihistamines for the preventive treatment of pollinosis.

**Objective:**

This study aimed to assess the efficacy of prophylactic treatment for nasal symptoms and acute asthma attacks by enrolling patients with *Artemisia* pollinosis to use a combined device of azelastine hydrochloride and fluticasone nasal spray prior to the pollen season.

**Methods:**

The study was registered at Chictr.org.cn (ChiCTR2300073758). A total of 120 patients with *Artemisia* pollinosis were randomly assigned to either a prophylactic treatment group or a control group at a 1:1 ratio. In the prophylactic treatment group, the nasal spray was initiated approximately two weeks before the onset of the pollen season.

**Results:**

During both the pollen season and the concurrent medication period, the prophylactic treatment group presented significantly lower total nasal symptom scores (TNSS) (means of 5.97 and 5.94) than the control group (means of 7.86 and 7.80) (*P* = 0.015 and 0.016). Although the prophylactic treatment group had a lower asthma attack rate than the control group, the difference was not statistically significant (*P* = 0.284).

**Conclusions:**

Prophylactic treatment with azelastine hydrochloride and fluticasone propionate nasal sprays can alleviate nasal symptoms and may reduce acute asthma attacks during the pollen season.

**Clinical Trial Registration:**

[Chictr.org.cn], identifier (ChiCTR2300073758).

## 1 Introduction

Pollinosis is caused by allergies to plant pollen, and patients often experience symptoms such as sneezing, conjunctival redness, and wheezing. These symptoms can significantly impair daily activities and quality of life. Pollinosis has become a common disease in European and American countries, and it is also increasing in some Asian regions. Baba and Matsubara et al. reported that the prevalence of Japanese cedar pollinosis increased from 16.2% in 1998 to 26.5% in 2008 and 38.8% in 2019, whereas the prevalence of pollinosis other than Japanese cedar pollinosis increased from 10.9% to 15.4% and 25.1%, respectively (1). A survey conducted by Wang Xueyan et al. in Inner Mongolia grassland in 2015 revealed that the average prevalence of AR in Inner Mongolia grassland was 32.4%, and the rate of pollinosis was as high as 18.5% (2). Given the burden of pollinosis (3), particularly in regions with high pollen counts, effective prophylactic treatments are urgently needed.

Previous studies have shown that the use of mometasone furoate aqueous nasal spray (4), fluticasone furoate nasal spray (5), and montelukast sodium (6) in advance for prophylactic treatment before the pollen season is helpful for relieving symptoms and increasing the number of asymptomatic days during the pollen season. In addition, if asthma patients are complicated with AR, the risk of needing emergency treatment, hospitalization or systemic use of glucocorticoids due to acute asthma attacks increases (7).

A fixed-dose combination of azelastine hydrochloride and fluticasone propionate (FDC-AzeFlu) delivered via one device is indicated for moderate-to–severe seasonal allergic rhinitis (SAR) and perennial allergic rhinitis. The advanced formulation of the nasal spray circumvents the need for individual administration of multiple drugs. In Asian countries, the use of FDC-AzeFlu is increasing, and in mainland China, it was approved and started to be used in 2022 (8). However, few studies have investigated FDC-AzeFlu for the prophylactic treatment of pollinosis.

In North China, *Artemisia* pollen is the most common and abundant pollen type and has the largest peak concentration of total autumn pollen according to daily airborne pollen monitoring (9, 10). *Artemisia* pollen had the highest positive specific IgE (sIgE) rate, up to 78.6%, in patients with autumn pollinosis in North China (11). In our study, patients with *Artemisia* pollinosis in Yulin city, Shaanxi Province, were selected to use FDC-AzeFlu in advance before the pollen season to evaluate the effect of prophylactic treatment on nasal symptoms and acute asthma attacks during the pollen season.

## 2 Materials and methods

### 2.1 Study population

According to previous literature reports, the average nasal symptom scores of the prophylactic treatment group (*n* = 116) and the control group (*n* = 115) during the pollen season were 0.7 and 1.8 respectively (*P* < 0.01) (4). The significance level *α* was set at 0.05 (two-sided), and the test power (1-β) was set at 0.8. It was estimated that the initial sample size for each group was approximately 54 cases. Considering the dropout rate of 10%, the adjusted sample size for each group was 60 cases. Therefore, a total of 120 *Artemisia* pollinosis individuals (51 males and 69 females) aged 12–65 years (mean age, 36.0 years) were recruited into the study. The participants were living in Yuli city and patients attending the First Affiliated Hospital of Xi’an Jiaotong University, Yulin Hospital (Yulin, Shaanxi, China). The inclusion criteria were as follows: (1) male or female aged 12 years and above; (2) AR was diagnosed through typical clinical symptoms (repeated sneezing, runny nose, stuffy nose or itchy nose) and signs, and the symptoms occurred mainly in autumn; and (3) The serum sIgE test for dust mites and molds was negative, while the *Artemisia* sIgE test was positive. Specific IgE values of 0.35 kU/L or greater were considered positive. The exclusion criteria were as follows: (1) individuals who have received or were currently undergoing allergen-specific immunotherapy; (2) receiving anti-IgE monoclonal antibodies or anti-IL4/IL13 monoclonal antibodies in the past six months or currently; (3) pregnant or lactating; and (4) having malignant tumors or other serious liver, kidney, heart, hematopoietic, neurological, or mental illnesses.

### 2.2 Study design

The eligibility and medical history of the 120 patients were assessed, after which they were randomly assigned to either a prophylactic treatment group or a control group at a 1:1 ratio using a computer-generated random sequence with sealed envelopes. To standardize the allergen testing method during the statistical process, measurements of sIgE for pollen (*Artemisia* and other tree, grass, and weed pollen), mites (*Dermatophagoides pteronyssinus* and *Dermatophagoides farinae*), and molds (*Alternaria alternata and Aspergillus fumigatus*) were retested by immunoblotting assays (Euroline, Medizinische LabordiagnostikaAG) at the initial visit on July 21. Autumn weed pollinosis was anticipated at the end of July or the beginning of August. Patients in the prophylactic treatment group started using the nasal spray on July 21, approximately two weeks prior. Meanwhile, upon exhibiting moderate or more severe nasal symptoms, the control group was instructed to begin using FDC-AzeFlu the following day. The observation period ended on September 25th, when the pollen season ended, and all patients stopped nasal spraying. The recommended dosage was 1 spray per nostril twice daily, and each spray contained 137 µg of azelastine hydrochloride and 50 µg of fluticasone propionate (Shufeimin®, Changfeng PharmTech, Inc.; Suzhou, China).

The present study was registered at Chictr.org.cn (ChiCTR2300073758). The ethical review was approved by the Institutional Review Board of the First Affiliated Hospital of Xi’an Jiaotong University, Yulin Hospital (2023014). Informed consent was obtained from each patient and the parents of all the participating children. Patients or the public were not involved in the design, or conduct, or reporting, or dissemination plans of our research.

### 2.3 Data collection and definition

All the patients were free of symptoms at the screening and kept electronic diaries from July 21 to September 25. Runny nose, nasal congestion, sneezing, and nasal itching were evaluated on a 4-point scale (0 = no symptoms, 1 = minimal, well-tolerated symptoms, 2 = bothersome but tolerated symptoms, and 3 = severe and hard to tolerate symptoms, causing interference with activities of daily living or sleeping). The total nasal symptom score (TNSS) was calculated as the sum of these individual symptom scores, ranging from 0 to 12 points (1–3 indicating mild symptoms, 4–12 indicating moderate to severe symptoms). Acute asthma attack is defined as the need for oral or intravenous administration of glucocorticoids, emergency treatment, or hospitalization due to asthma. In parallel, daily pollen counts were performed throughout the study via a 7-day volumetric spore trap (Burkard Scientific Equipment, Hertfordshire, England). The pollen monitoring point was located at Yulin Hospital, which was located in the center of Yulin city.

### 2.4 Statistical analysis

Categorical data were compared via the *χ*^2^ test. ANOVA was used to analyze continuous data. The TNSS between groups were compared via repeated-measures ANOVA. The Pearson correlation coefficient was used to measure the linear relationship between two continuous variables. Statistical analyses were performed with SPSS version 13 (SPSS Inc., Chicago, USA).

## 3 Results

Among the 120 participants with *Artemisia* pollinosis, 3 withdrew from the study because of poor compliance, and 10 were not included in the analysis because of incomplete data. Therefore, 107 patients were documented with complete data for atopy and provided full data during the follow-up period. The demographic characteristics (age and sex) of the patients in the prophylactic treatment group and the control group were comparable (Table 1). Patients in both groups had similar histories of AR and asthma (Table 1), and there were also no significant differences between the groups regarding atopy (Table 1).

**Table 1:** Characteristics of the patients in the study.

Characteristics	Prophylactic treatment group (*n* = 54)	Control group (*n* = 53)	*P* value
Age, y, mean ± SD	37.2 ± 9.73	35.0 ± 13.54	0.327
Sex, *n* (%)			
M	24 (44.4%)	18 (34.0%)	0.267
F	30 (55.6%)	35 (66.0%)	
Duration of AR, y, Mean ± SD	10.8 ± 9.73	10.2 ± 9.73	0.595
Diagnosis of asthma, *n* (%)	2,342.6%	2,037.7%	0.608
Family history of allergies, *n* (%)	814.8%	1,120.8%	0.421
sIgE levels with *Artemisia*, *n* (%)			
1 (0.35–0.7 kU/L)	5 (9.3%)	7 (13.2%)	0.775
2 (0.7–3.5 kU/L)	11 (20.4%)	12 (22.6%)	
3 (3.5–17.5 kU/L)	10 (18.5%)	13 (24.5%)	
4 (17.5–50 kU/L)	18 (33.3%)	14 (26.4%)	
5 (50–100 kU/L)	10 (18.5%)	7 (13.2%)	

**Figure 1:**
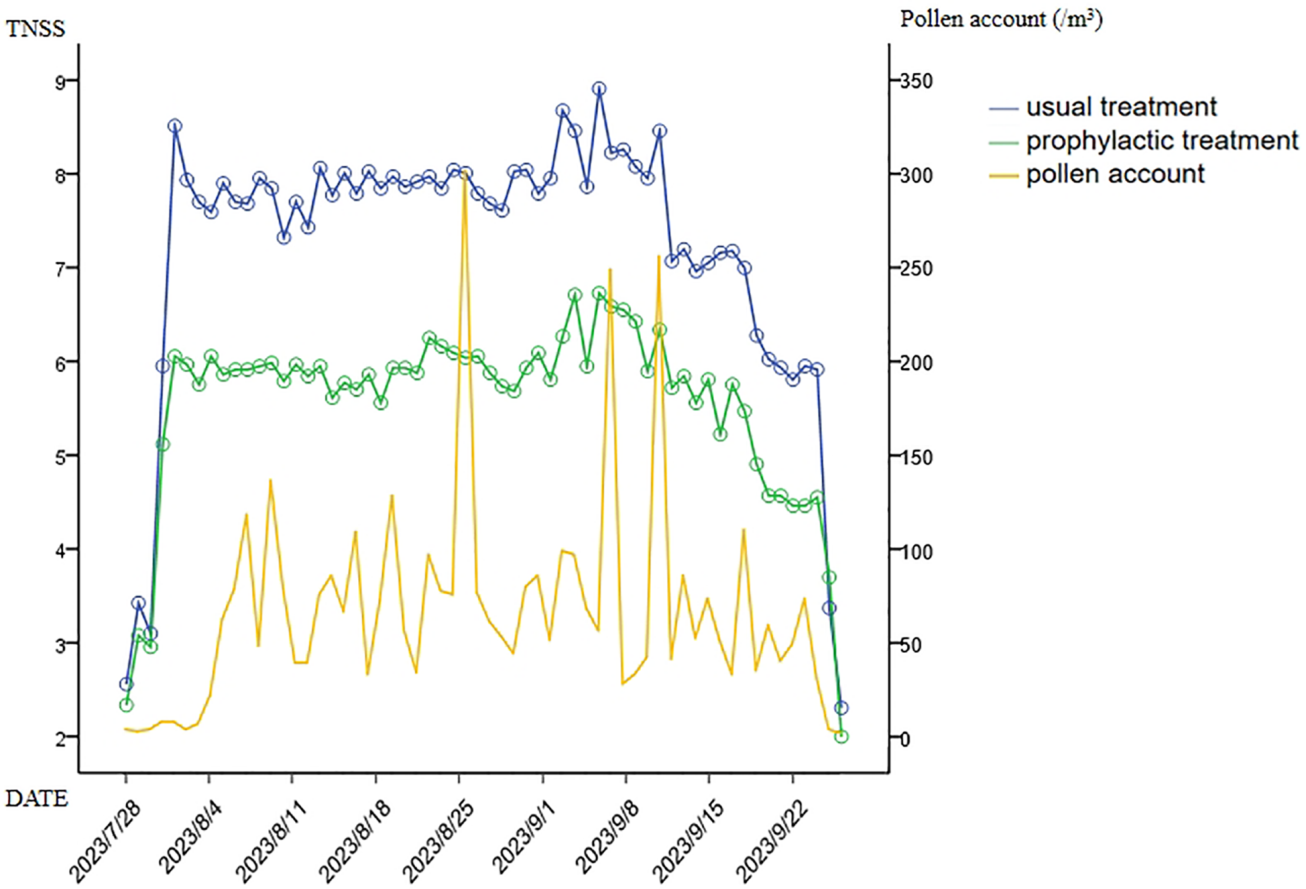
The TNSS of the control group and the prophylactic treatment group were positively correlated with pollen concentration, with Pearson correlation coefficients of 0.386 and 0.385, respectively. The daily TNSS was significantly lower in the prophylactic treatment group during both the pollen period (from August 4th to September 23rd) and the concurrent medication period (from August 1st to September 24th).

The pollen count results are shown in Figure 1. According to pollen monitoring, the pollen concentration began to increase on August 4th (24 /m^3^) and then fluctuated within the range of 30–137 /m^3^. There were three thunderstorms and strong winds on August 25th, September 6th, and September 10th, causing a sharp increase in the pollen concentration. On September 24th, the pollen concentration significantly decreased to 6 /m^3^. On July 31st, the nasal symptoms of the control group significantly worsened, and the TNSS score increased to 6.13. On August 1st, the nasal spray was distributed, and medication was started.

During both the pollen period (from August 4th to September 23rd) and the concurrent medication period (from August 1st to September 24th), the prophylactic treatment group presented significantly lower TNSS (mean, 5.97 and 5.94) than the control group (mean, 7.86 and 7.80) (*P* = 0.015 and 0.016) (Figure 1). Furthermore, the use of additional medications (antihistamines and antileukotrienes) for breakthrough nasal symptoms was slightly less common for the prophylactically treated patients, but this difference was not statistically significant (Table 2).

**Table 2:** Rates of use of additional medication during the pollination period.

Group	No use of additional medication, *n* (%)	Use of additional medication, *n* (%)	*P* value
Control group	19 (35.8%)	34 (64.2%)	0.198
Prophylactic treatment group	26 (48.1%)	28 (51.9%)	

During the pollination period, 41 (38.3%) participants experienced asthma attacks. The prophylactic treatment group had a lower asthma attack rate than the control group, but this difference was not statistically significant (Table 3).

**Table 3:** Rates of asthma attacks during the pollination period.

Group	Non asthma attack, *n* (%)	Asthma attack, *n* (%)	*P* value
Control group	30 (56.6%)	23 (43.4%)	0.284
Prophylactic treatment group	36 (66.7%)	18 (33.3%)	

## 4 Discussion

Pollen syndrome is an inflammatory reaction caused by the inhalation of corresponding pollen allergens into the respiratory tract, and the concentration of pollen allergens is a key factor in the onset of pollinosis symptoms. Ouyang Yuhui et al. reported that *Artemisia* pollen had a small pollen dispersal period of approximately 2 weeks before the pollination period, followed by a peak period of 4 weeks (12). The amount of *Artemisia* pollen during the peak period accounted for 76.2% of the total. The nasal mucosa of patients with pollinosis can have the lightest persistent inflammatory reaction because of the continuous stimulation of allergens during the period of small amounts of pollen scattering (13). Canonica proposed minimal persistent inflammation in AR; that is, the nasal mucosa of AR patients consistently exhibits mild allergic inflammation (13). Storms et al. reported that inflammatory infiltration occurred in the nasal mucosa of SAR patients without symptoms (14). Studies have shown that early intervention therapy plays an important role in improving nasal mucosal inflammation, to some extent by inhibiting the expression of intercellular adhesion molecule 1 (ICAM 1) and the infiltration of eosinophils (15). Consistent with previous studies investigating prophylactic treatments using mometasone furoate aqueous nasal spray, fluticasone furoate nasal spray, and montelukast sodium, our study demonstrates that prophylactic treatment with FDC-AzeFlu effectively alleviates nasal symptoms in patients with *Artemisia* pollinosis. However, due to differences in study populations and sensitizing allergens, direct comparisons with the preventive medications used in previous studies are challenging (4–6).

There are no definite suggestions for specific drugs or period for the prophylactic treatment of SAR. Generally, prophylactic treatment starts 2–4 weeks in advance and lasts for 4–8 weeks until the end of the pollen period (16). In the early stage of pollen pollination, that is, two weeks before the peak of pollen dispersal, we treated patients with FDC-AzeFlu. The results revealed that, compared with those of the control group, the TNSS of patients in the prophylactic treatment group were lower. Our study did not restrict the use of additional medications, such as antihistamines and antileukotrienes, for breakthrough nasal symptoms, which might bias the results. However, the control group used more additional medication. Therefore, this bias does not affect the results of this study.

Previous studies (Italy, Canada, China) reported the occurrence of seasonal asthma and increased bronchial responsiveness from seasonal pollen exposure in patients with pollinosis (17–19). AR combined with asthma can increase the incidence of asthma-related events, such as asthma attacks and emergency visits (7). AR increases the dosage of medication for emergency treatment in asthma patients (20). The combination of inhaled and nasal steroids can significantly reduce the risk of asthma-related emergency visits in asthma patients with rhinitis (21). Nasal steroids combined with antihistamines can effectively reduce lower respiratory tract inflammation in patients with SAR during the pollen season (22).

Our study revealed that the asthma attack rate was lower in the prophylactic treatment group than in the control group, but this difference was not statistically significant. The possible explanations are as follows: (1) the influence of sample size, which may be statistically significant after the sample size is increased appropriately; (2) asthma, which refers to the acute attack of respiratory diseases such as asthma caused by thunderstorm weather (23, 24). The symptoms are serious, and the disease develops rapidly, usually manifesting as a group of patients experiencing simultaneous asthma attacks. In this study, thunderstorms were recorded on September 2, September 10 and September 17, accompanied by a short-term sharp increase in pollen concentration, and patients may have developed asthma.

Our study had several limitations. How prophylactic treatment with FDC-AzeFlu might influence local inflammation, such as inflammatory cell infiltration and the expression of cytokines in nasal secretions, has not been thoroughly investigated. No significant differences in asthma attack rates were observed in this study. Beyond augmenting the sample size, the incorporation of peak expiratory flow monitoring may provide valuable insights into airway resistance. It is recommended that future studies include these critical data to provide a more comprehensive analysis.

## 5 Conclusion

For patients with *Artemisia* pollinosis, prophylactic treatment with FDC-AzeFlu can alleviate nasal symptoms and potentially reduce acute asthma attacks during the pollen season.

## Data Availability

The raw data supporting the conclusions of this article can be obtained from the authors upon reasonable request.
